# Social correlates of the dominance rank and long-term cortisol levels in adolescent and adult male rhesus macaques (*Macaca mulatta*)

**DOI:** 10.1038/srep25431

**Published:** 2016-05-05

**Authors:** Xiaoli Feng, Xujun Wu, Ryan J. Morrill, Zhifei Li, Chunlu Li, Shangchuan Yang, Zhaoxia Li, Ding Cui, Longbao Lv, Zhengfei Hu, Bo Zhang, Yong Yin, Liyun Guo, Dongdong Qin, Xintian Hu

**Affiliations:** 1 Key Laboratory of Animal Models and Human Disease Mechanisms of Chinese Academy of Sciences & Yunnan Province, Kunming Institute of Zoology, Kunming, Yunnan, 650223, China; 2 Kunming College of Life Science, University of Chinese Academy of Sciences, Kunming, Yunnan, 650204, China; 3 State Key Laboratory of Brain and Cognitive Science, Beijing, 100101, People’s Republic of China; 4 Kunming Primate Research Center, Kunming Institute of Zoology, Chinese Academy of Sciences, Kunming, Yunnan, 650223, China; 5 Institute of Biophysics, the Chinese Academy of Sciences, Beijing, 100101, People’s Republic of China; 6 CAS Center for Excellence in Brain Science, Chinese Academy of Sciences, 320 Yue Yang Road, Shanghai, 200031, China; 7 Department of Rehabilitation Medicine, the Fourth Affiliated Hospital of Kunming Medical University, Kunming, Yunnan, 650021, China; 8 The Ophthalmology Department, the Fourth Affiliated Hospital of Kunming Medical University, Kunming, Yunnan, 650021, China

## Abstract

A common pattern in dominance hierarchies is that some ranks result in higher levels of psychosocial stress than others. Such stress can lead to negative health outcomes, possibly through altered levels of stress hormones. The dominance rank-stress physiology relationship is known to vary between species; sometimes dominants show higher levels of glucocorticoid stress hormones, whereas in other cases subordinates show higher levels. It is less clear how this relationship varies between groups of different ages or cultures. In this study, we used long-term cortisol measurement methods to compare the effect of rank on cortisol levels in adult and adolescent male rhesus macaques. In the adult groups, subordinates had significantly higher cortisol levels. In the adolescents, no significant correlation between cortisol and status was found. Further analysis demonstrated that the adult hierarchy was stricter than that of the adolescents. Adult subordinates received extreme aggression more frequently than dominants, and this class of behavior was positively correlated with cortisol; by contrast, adolescents showed neither trend. Together, these findings provide evidence for a cortisol-rank relationship determined by social factors, namely, despotism of the group, and highlight the importance of group-specific social analysis when comparing or combining results obtained from different groups of animals.

For social animals, apart from humans, rank within a dominance hierarchy can be a source of chronic stress^1^,^2^. Holding a stressful rank is thought to result in sustained activation of the neuroendocrine “fight or flight” response, which in turn results in long-term physiological and metabolic alterations and often increased rates of disease and mortality^3^,^4^,^5^. The oft-noted socioeconomic status (SES) health gradient, in which individuals with a higher SES have better health outcomes than those below them, is thought to be due, in part, to psychosocial stress from the hierarchy^3^,^6^,^7^. This hypothesis draws support from an extensive literature showing associations between dominance ranks, levels of the glucocorticoid stress hormone cortisol, and health outcomes in nonhuman primates (NHPs). However, the NHP literature also suggests that whether dominance or subordinance is more stressful varies among different species. A meta-review of NHP studies showed that some species exhibited higher cortisol concentrations in dominants than subordinates (female common marmosets and male and female cotton top tamarins), whereas others demonstrated higher cortisol levels in subordinates than dominants (female cynomolgous monkeys, male squirrel monkeys, male olive baboons and male talapoins) and still others reported approximately equal cortisol levels in dominants and subordinates (female squirrel monkeys and female talapoins)^1^. Social rank and stress physiology patterns have even been shown to vary between populations of the same species. For instance, cortisol levels among subordinate olive baboons (*Papio anubis*) appear to be a function of dominance styles and the prevalence of prosocial behavior, which are population-specific^8^. Thus, no consensus exists concerning the relationship between hierarchy and stress levels. Despite the heterogeneities, a common pattern is that the most physically and psychologically stressed individuals have higher rates of stress-related pathologies^2^. Thus, aspects of social organization that putatively determine rank-associated physiology in NHPs may also represent important determining factors for human susceptibility to psychosocial stress-related disease.

A set of social organization factors to predict rank physiology patterns was outlined by Sapolsky^2^ and previously used to interpret rank-cortisol variations between different groups^9^. Most relevant to the present study is the manner in which dominance is maintained in a hierarchy. According to the prediction, dominant individuals in societies where dominance is maintained through frequent physical aggression exhibit higher physiological stress indices due to the physical demands of fighting. Conversely, subordinates in hierarchies in which dominants maintain status through psychological intimidation are coerced into frequent displays of submission. Thus, they have higher physiological stress indices due to their vulnerability to more attacks by other animals, especially if they have few outlets to address the conflict stressors and a lack of alternative strategies to hierarchy competition (e.g., social support), both of which might help cope with high pressure^2^. Additional factors include coping outlets for subordinates, the level of competition for mates, the ability of subordinates to avoid dominants, and even individual personalities and cultures^2^. Reduction of rank-physiology variability to quantifiable social organization factors may have clinical potential for the evaluation of stress-related disease risks, but questions about these hypotheses remain. In particular, variations between age classes of the same species have received relatively little attention despite readily notable age differences in social organization and behavior^10^,^11^. Furthermore, little is known about the social characteristics of adolescent hierarchies. Thus, although numerous Old World primate species acquire their dominance rank early in life^12^,^13^,^14^, adolescent social behavior suggests that their rank physiology may vary from that of adults.

One of the few studies addressing adolescent rank physiology examined male rhesus macaque (*Macaca mulatta*) monkeys and concluded that rank was not correlated with plasma cortisol levels in adolescents^15^; however, this study did not present comparable data for adults. Furthermore, potentially conflicting results have been published regarding rank physiology relationships in adult male rhesus monkeys. One study found no correlation between social rank or levels of aggression and cortisol in captive adult macaques^16^. However, another study of free-ranging monkeys suggested that high levels of aggression, which were commonly associated with social dominance, positively correlated with cortisol^17^. Differences in rank physiology may be due to inter-population variability, although further research focusing on dominance rank and stress is needed before any conclusions can be made.

Importantly, methodological confounds in the above-mentioned studies cannot be ruled out. The only published data on male rhesus macaque rank physiology utilized plasma measurements^15^,^16^,^17^. This method typically requires stressful procedures such as capture, restraint and venipuncture, which can elevate circulating cortisol levels quickly and is also subject to circadian effects, both of which confound reliable measurement^18^. For the study of rank physiology, measurements of stable and long-term hypothalamic pituitary axis activity are desirable. Measurement of cortisol from hair samples represents the long-term cumulative secretion of active cortisol and is considered more reliable than methods such as blood, fecal or urine sampling to assess baseline cortisol levels^18^,^19^.

In this study, we investigated the relationship between dominance rank and stress physiology separately in adult and adolescent populations of male rhesus macaques using measurements of cortisol from hair samples. Additionally, we calculated hierarchy linearity and recorded behaviors from each age class to examine potential social and behavioral factors influencing the rank physiology patterns.

## Results

### Hierarchy linearity

The H values for the measurement of the strictness of social ranks for the 2 adult groups were 0.80 and 0.77, whereas the H values for the 2 adolescent groups were 0.68 and 0.53. H values vary between 0 and 1, with a higher H value indicating a much stricter social rank. If the H value of a society is closer to 1, then the rank of this society is more despotic and the individual with the highest rank holds the best access to resources. If the H value of a society approaches 0, then its hierarchy is less stringent and the access to resources for each individual within the society is relatively equal. Thus, the adult society was more despotic than the adolescent society.

### The relationship between dominance and cortisol in adults and adolescents

**Figure 1:**
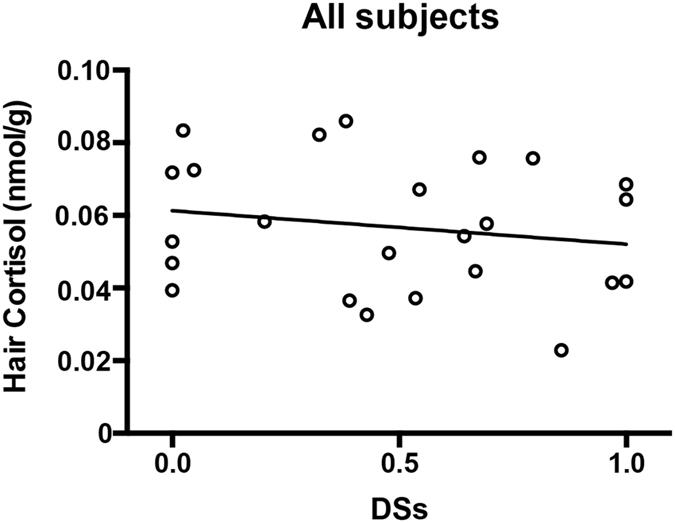
Correlation between DS_S_ (as a marker of social status) and hair cortisol levels in all males (r = −0.183, p = 0.393).

**Figure 2:**
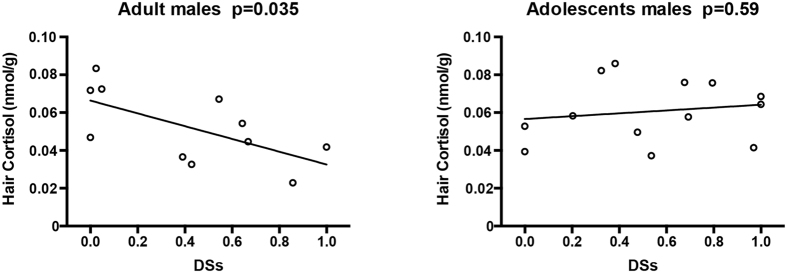
Relationships between DS_S_ (as a marker of social status) and hair cortisol in adults (Fig. 2A; r = −0.636, p = 0.035) and adolescents (Fig. 2B; r = 0.163, p = 0.595).

When we analyzed the relationship between social rank and cortisol among all the males, no significant correlation was discovered (r = −0.183, p = 0.393; Fig. 1). However, a unique correlation between rank and cortisol levels for the adult groups emerged after dividing all 24 male monkeys into 2 groups according to age (Fig. 2). Correlation analysis indicated that rank was significantly inversely correlated with cortisol levels in adults (Fig. 2A; r = −0.636, n = 11, p = 0.035). In contrast, a significant relationship did not exist among adolescents (Fig. 2B; r = 0.163, n = 13, p = 0.595).

### Behavior patterns in adults and adolescents

**Figure 3:**
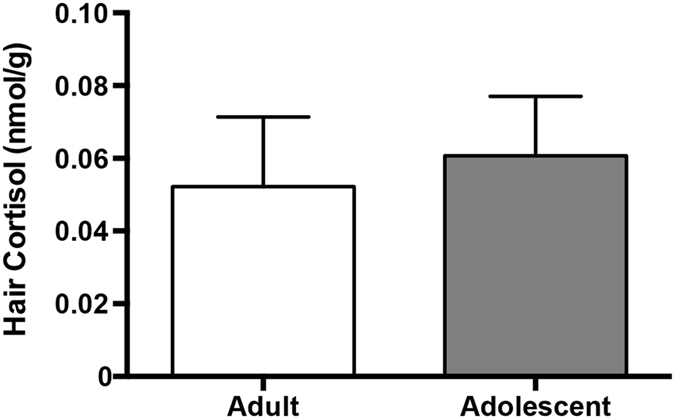
Differences in hair cortisol levels between adults and adolescents (t-test, p = 0.254).

Because no age-specific difference existed in hair cortisol levels between adults and adolescents (Fig. 3, t(11,13) = 1.171, p = 0.254), we determined potential behavioral correlates of the varying relationships of status and cortisol between the two different age groups. We hypothesized that, of the eight stress-related classes of behaviors measured for rank determination (receipt of extreme aggression, receipt of mild aggression, receipt of extreme submission, receipt of mild submission, extreme aggressive displays, mild aggressive displays, extreme submissive displays and mild submissive displays), receipt of aggression or submissive displays should correlate with cortisol because these categories represent an individual’s psychological appraisal of a stressor^20^.

**Figure 4:**
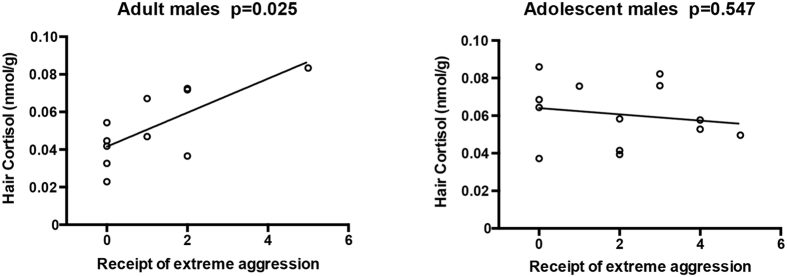
Correlation of the receipt of extreme aggression to hair cortisol (r = 0.686, p = 0.025, adult; r = −0.160, p = 0.547, adolescent).

**Figure 5:**
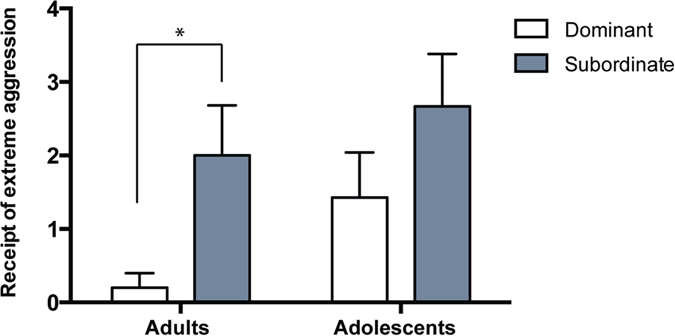
Comparison of the rate of receipt of extreme aggression between dominant and subordinate adults and adolescents (Mean ± SEM). (t(5,6) = 2.318, p = 0.046, adults; t(6,7) = 1.325, p = 0.212, adolescents).

Among adolescents, none of the rates of the analyzed behaviors were correlated with hair cortisol levels (Table 1). Among adults, receipt of extreme aggression (but not receipt of mild aggression) was positively related to hair cortisol (Fig. 4, Table 2; r = 0.686, n = 11, p = 0.025). Furthermore, receipt of extreme aggression in subordinate adults was significantly higher than in dominant adults (t(5,6) = 2.318, p = 0.046), as shown in Fig. 5. Thus, the receipt of extreme aggression may be used as a behavioral measurement of stress. Significant differences did not exist among subordinate and dominant adolescents, suggesting a more equitable distribution of stress among this age group.

The receipt of extreme aggression did not differ significantly in dominant adults and dominant adolescents (F(1, 10) = 2.666, p = 0.134) or in subordinate adults and subordinate adolescents (F(1, 10) = 0.455, p = 0.515).

Additionally, adolescents showed a trend of negative correlation between social contact bsehavior and long-term cortisol levels (r = −0.536, p = 0.059).

## Discussion

In the present study, we examined the rank physiology of single-sex, laboratory-housed adult and adolescent rhesus monkeys. Upon finding no relationship between rank and cortisol, we separated the rhesus monkeys into different age groups and discovered two distinct trends with distinct socio-behavioral correlates (i.e., rank correlated negatively with cortisol in adults but no significant correlation of rank and cortisol was determined in adolescents). Behaviorally, adult subordinates received extreme aggression more than dominants and the frequency of this behavior was correlated with long-term cortisol levels. Neither trend was observed in adolescents.

Although the receipt of extreme aggression did not differ significantly in dominant adults and dominant adolescents or in subordinate adults and subordinate adolescents, this type of behavior positively predicted long-term cortisol levels in adults but not in adolescents. This result indicates that the receipt of extreme aggression by subordinate individuals may be the cause of elevated stress among adults.

Moreover, the H values of the adult groups were higher than those of the adolescents, suggesting that the adult hierarchy was stricter than that of the adolescents.

Taken together, these results suggest that the style of dominance maintenance differed between the adult and juvenile populations and was more stressful psychologically for adult subordinates. The despotism of adult rhesus monkey culture results in increased psychosocial stress for subordinates and in turn a sustained elevation of HPA activity. Likewise, the less despotic adolescent culture results in indistinguishable cortisol levels for subordinates and dominants. In line with Sapolsky’s predictions, variations in the strictness of the hierarchy and the manner in which dominance is maintained appear to be determining factors in rank physiology^2^.

If the rank physiology relationship is determined by the degree of despotism, what factors determine this cultural facet? On the level of social organization, dominants have been observed to become relatively more stressed than subordinates in hierarchies undergoing reorganization due to the increased physical violence required for the maintenance of rank. Because dominants vie for power, their focus shifts away from coercing submission from lower-ranking individuals. When stability returns, subordinates again show elevated physiological stress indices^21^. Another explanation for the unique significant correlation between ranks and cortisol levels in adults but not in adolescents is that dominance hierarchies may also undergo organizational strengthening through maturation. Specifically, for adolescents in a stable and long-lasting environment, stress is distributed more equally within the society and thus the cortisol levels are not significantly different between dominants and subordinates. However, for adults in a similarly stable living environment, the linearity of the society has changed into a more despotic one compared to that of the adolescents. As a result, the subordinates have to bear more stress than their dominant counterparts and show elevated cortisol levels, which may indicate that dominance hierarchies undergo organizational strengthening through maturation as adolescents grow into adulthood.

Sociobehavioral coping strategies, such as high levels of grooming and social contact, are another factor hypothesized to suppress physiological stress indices in stress-prone ranks^1^,^2^. Social contact might influence the cortisol levels of the participants by relieving the stress induced by the hierarchy competition or the aggressive encounters; therefore, this factor is important in NHP and human studies because both groups are highly dependent on social life. In our study, adolescents showed significantly higher levels of correlation between social contact behavior and long-term cortisol levels. Even so, we still believe it is important to take social contact or other social affiliative behaviors into account when determining stress levels, which might represent an important avenue of study in both NHP models and humans.

Although rank-related phenomena are easier to study in NHPs, humans are also thought to be vulnerable to psychosocial stress stemming from rank in the SES hierarchy^2^,^7^. As with NHPs, it is thought that such stress may become pathological for humans. Low SES individuals generally experience worse health outcomes than those above them, including increased risks of cardiovascular, respiratory and psychiatric diseases and increased overall mortality^22^,^23^,^24^. These health inequalities do not exist solely below the poverty threshold but show a gradient throughout the entire SES hierarchy, with those at the highest SES levels having better health outcomes than those immediately below them^3^,^6^. Moreover, perceived stress during adolescence may have a long term influence on adult health^25^. This finding suggests that physical factors alone, such as access to healthcare or unsanitary living conditions, cannot explain the gradient. Likewise, animal research shows that high levels of stress hormones are associated with negative health sequelae such as coronary artery disease^4^, depressive behavior^5^ and hypothalamic pituitary axis dysfunction^26^,^27^. Research on both humans and animals supports a role for psychosocial stress in the SES-health gradient. Therefore, there has been considerable interest in the development of rank-stress models to increase our understanding of and develop therapeutics for human stress-related diseases^5^,^28^.

While epidemiological research shows that SES is generally inversely related to stress and health, few studies have examined rank relationships between groups that vary in social structure and culture. The question of how SES or other hierarchical social organizations affect adolescent groups stands out. In one of the few studies to address this question, West *et al.* recently showed that cortisol levels in secondary school students did not vary with family SES^9^. Instead, hierarchies predictive of cortisol levels were adolescent-specific (i.e., scholastic, sports-related and social hierarchies). Furthermore, rank physiology patterns varied between hierarchies and gender. For example, cortisol levels in males increased in a gradient as social hierarchy status increased, whereas in females only the “top” levels of the hierarchy showed elevated cortisol. This work suggests that humans, like NHPs, exhibit variability in rank physiology patterns depending on their age group.

Another human study of adolescents found that status had a significant effect on salivary cortisol levels among females but not males, which was similar to the result in the present study^29^. However, other researchers have found that lower long-term cortisol levels were associated with low social stress in 85 male adolescents^30^. In a group of subjects with developing depression, individuals showed decreased cortisol levels when their social evaluation was lower^31^. Moreover, researchers reported that the sensitivity of the HPA axis (the end product of the HPA axis is cortisol in primates) was enhanced as age increased^32^,^33^, whereas we found no significant difference in cortisol levels in adults and adolescents.

Possible explanations for the difference in the findings of these studies and the present study on the relationship between cortisol levels and social status might include the following items. First, previous studies focused primarily on either an acute cortisol response to social stress or cortisol alterations in the context of neuroendocrine disorders (e.g., depression), whereas this study examined long-term cortisol levels under normal circumstances. Second, previous studies utilized plasma, saliva or urine samples to measure cortisol levels. These methods were useful for assaying acute cortisol levels that only reflected short-term stress that occurred over hours to days and could not assess chronic stress levels that occurred over a period of weeks to months without repeated sampling of the animals. In contrast, cortisol levels measured from hair can reflect chronic stress^19^,^34^. In this study, cortisol levels were measured from hair grown during the behavioral sampling period and therefore represented the mean cortisol production associated with the chronic stress experienced by the animals.

Here, we show that the dominance rank-physiology relationship varies between groups of an individual species and that this variability is determined by differences in the despotic tendencies of each group. Long-term hair cortisol displays an inverse relationship with the dominance rank for adults, whereas no significant relationship exists for adolescents. We demonstrate that the adult hierarchy is more linear than the adolescent hierarchy. To the best of our knowledge, this is the first demonstration of age differences in rank physiology using methods designed for the measurement of long-term baseline cortisol levels. In light of the proposed hypothesis that rank-physiology can vary between species and even populations, the findings presented here should not be overgeneralized. Instead, consideration of the diversity of NHP social systems and their associated hierarchies will allow for the development of social stress models that address nuances in human social organization.

The animals in the present study were all male. Another study by our team demonstrated that cortisol levels were not correlated with social rank in despotic groups of female adult monkeys (one social group consisted of one male and 4 or 5 female monkeys), but a negative correlation was found between social rank and cortisol levels in less stringent hierarchies^35^. The inconsistency might due to the different animal sex or social organizations from those analyzed in the present study.

Moreover, unisex groups of adult males (the construct adopted in this study) do not exist in free-living groups of macaques. This artificial construct may contribute to the extreme despotic nature of the hierarchies in single-sex adult groups and the relationship between behavior and cortisol levels. Hence, studies on mixed-sex adolescent monkeys might be of great importance and would be enlightening for our future work.

## Methods

### Subjects

The adult subjects were 11 male rhesus macaques (*Macaca mulatta*) aged 6–10 years that were housed in 2 isosexual social colonies (n = 7 and 4). The adolescent subjects were 13 male rhesus macaques aged 4–5 years that were housed in 2 isosexual social colonies (n = 7 and 6). The monkeys were housed in colonies (3 × 5 × 3 m) with indoor/outdoor access. All animals had continuous access to water and monkey chow supplemented with seasonal fresh fruit or vegetables. The animals had lived in their respective social groups for at least 5 months prior to the initial sampling. All animal procedures were conducted in accordance with the National Institutes of Health Guide for the Care and Use of Laboratory Animals and were approved by the Biological Research Ethics Committee of the Kunming Institute of Zoology, Chinese Academy of Sciences.

### Experimental design

Behavior was video recorded for later analysis of social rank using a focal follow technique^36^, which is described below in the Behavioral Analysis section. After completion of the recording, hair samples were gathered. An individual’s rank in the hierarchy was determined post hoc through the outcomes of agonistic dyadic interactions recorded on videotape. Based on these interactions, the linearity of the hierarchy (H values for the social groups) was calculated. Finally, the correlation between status and long-term cortisol was calculated.

### Hair cortisol extraction

The assessed hair samples accumulated cortisol over the previous 3–6 months. Hair has a fairly predictable growth rate of approximately 1 cm per month^35^. Thus, the most proximal 1 cm segment to the skin approximates the latest month’s cortisol production, the second most proximal 1 cm segment approximates cortisol production during the month before the last month and so on^34^,^37^.

Prior to hair sampling, each animal was briefly anesthetized with ketamine HCl (15 mg/kg IM) and then moved out of his colony. A sample of hairs 4–5 cm long was clipped from the posterior vertex region of the neck between 1500 and 1600. The procedure for extracting hair cortisol has been described in detail in previous papers^18^,^19^. Briefly, the hair samples were washed twice for 3 min each time in 5 ml of isopropanol to remove surface contaminants, dried at 37 °C for 8 hours, and then pulverized by a ball mill (MM400, Retsch Inc.) at 26 Hz for 2.5 min. Approximately 500 mg of the powdered hair was weighed and incubated in 10 ml of methanol at room temperature for 24 hours with slow rotation to the extracted cortisol. Thereafter, the sample was centrifuged at 8000 G for 5 min and 4 ml of the supernatant was pipetted into a centrifuge tube. Then, the sample was dried under a stream of nitrogen gas. Finally, the extract was reconstituted by adding 0.5 ml of phosphate buffer solution and stored at −20 °C for later analysis. A total of 24 samples were obtained and cortisol was extracted from each sample. Hair cortisol concentrations were analyzed using a radioimmunoassay kit designed for cortisol determination (Cortisol RIA kit, REF IM1841, Beckman Coulter). The detection limit of cortisol was 1 × 10^−5^ nmol/mg. Intra-assay variations and cross-reactivity were separately listed in the tables below. We tested all hair samples using kits from the same series to eliminate the issue of inter-assay variation. Moreover, we tested the same sample twice to obtain the average value as the final cortisol level. Specifically, the sample from one monkey was divided into 2 equal portions prior to the test; these portions were tested separately and simultaneously, thereby yielding two replicates for the monkey. Then, we averaged the two values to obtain the final cortisol level of the monkey. Intra-assay variation information provided with the kit showed that the CV varied from 3.1–5.8%. Cross-reactivity of the main steroids that might cross-react with the antibody varied from 0.1% to 18%. Importantly, the kit was initially designed for serum cortisol and urine cortisol detection. Thus, the cross-reactivity of the steroids reflected the real values when the samples were blood or urine.

### Behavior sampling

Prior to sampling, a period of 5–7 days was assigned to familiarize the monkeys with the observers and cameras. A digital video camera fixed on a tripod was set up in front of the colony for the focal recording of one of the monkeys in the cage. Observers stayed far away from the cage (approximately 5 meters) during the recording to avoid disturbing the animals. Two records were collected from each monkey per day (30 min in the morning and 30 min in the afternoon). A total of 6 records were obtained from each monkey. Behavioral records were obtained during a 3-month period. We recorded twice per day from different monkeys—once for an adult and once for an adolescent. All 6 records for each monkey consisted of 3 records obatined in the morning and 3 in the afternoon.

For the evaluation of the frequencies of the behaviors of interest in each record, the videos of the focused monkeys were randomized and coded by three technicians. The three viewers examined each video simultaneously and came to a consensus about behavior classification. All viewers were blinded to the cortisol levels of the subject animals during the behavior coding.

### Social rank determination

The social status of each monkey was determined based on the consistent outcomes of aggressive encounters. Extreme aggression included bites, grabs, and slaps. Mild aggression included the stare threat, open-mouth threat, chase, and displace. Extreme submission included screams, crouches, screaming threat, and fleeing. Mild submission included lip smacks, grimaces, submissive presents, and moving away^5^. Each of the above conflict behaviors was scored as either initiating (performing) or receiving (from another monkey), and the frequencies were calculated per minute.

Based on the averaged frequencies, we determined the hierarchy and assigned each individual a rank using the David’s score (DS) method, described in detail elsewhere^38^. Briefly, the DS for each member *i* in a group was calculated using Equation 1: DS = w + w_2_−l−l_2_, where w represented the sum of *i*’s *P*_*ij*_ values (*P*_*ij*_ represented the proportion of wins by individual *i* in its interactions with another individual *j*), w_2_ represented the summed w values (weighted by the appropriate *P*_*ij*_ values) of the individuals with which *i* interacted, l represented the sum of *i*’s *P*_*ji*_ values (*P*_*ji*_ represented the proportion of wins by individual *j* in its interactions with another individual *i*) and l_2_ represented the summed l values (weighted by the appropriate *P*_*ji*_ values) of the individuals with which *i* interacted. For the standardization of DS values (DS_S_), the smallest DS value (the largest negative value) in a group was added by its absolute value to yield zero; then, the other DS values in the same group were added to the same value. The new DS values were divided by the largest new DS value. For each group, the DS_S_ of the highest rank was 1 and the lowest rank was 0. Within a social group, when the DS_S_ for an individual was less than 0.5, the monkey was considered subordinate; otherwise, it was considered dominant.

To measure the linearity of the social hierarchy of a group, the H value was calculated using Equation 2 as previously described^39^: H = [12/(n^3^ – n)] 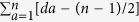 ^2^, where 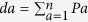 , *Pa* referred to the proportion of encounters won by the focused monkey against another in a pair-wise encounter, and *n* referred to the number of members in a social group. The H value varied from 0 to 1, with 0 indicating no definite pattern of outcomes in agonistic interactions in the social group and 1 indicating a linear ranking from most subordinate to most dominant; that is, a higher H value indicated a more despotic and highly linear group.

### Social contact determination

Social contact behavior referred to any body contact and close sitting of two individuals in a group. The duration and frequency of this type of behavior was recorded and analyzed.

### Behavioral analysis

Although several monkeys may have appeared in a recorded video, we concentrated on only one monkey and analyzed its behaviors. The specific procedures of behavioral measurements are as follows. Using the social contact behavior of Monkey A (the focused monkey) as an example, when Monkey A started its social contact behavior in the video, the video was paused manually and the precise time point displayed on the screen was recorded. Next, the video was resumed until the social contact stopped and the precise time point was again recorded. The period (in seconds) between the two time points was considered one episodic event of social contact. Many episodes of social contact might occur in a 30-min video. The duration of social contact was calculated by adding all episode durations, and the number of episodes was counted as the frequency of social contact. Afterwards, the duration and frequency of social contact obtained from all recorded videos of this monkey were summed and then divided by the total time (in minutes, 6 × 30 = 180 min total) of the behavioral recordings of the monkey, which yielded two values in s/min (duration) and times/min (frequency) for later analysis. The other behaviors of the focused monkey were analyzed similarly.

### Statistical analyses

The Pearson correlation was used to analyze the relationship between hair cortisol levels and social status among all males (adults and adolescents). Student’s t-test was used to compare hair cortisol concentrations between adult and adolescent males and the rates of behaviors between subordinate and dominant males. In the investigations of relevance between behaviors and hair cortisol, the non-parametric Spearman’s rank correlation was conducted. All tests were two-tailed unless otherwise noted. Probabilities < 0.05 were considered statistically significant. All analyses were conducted in R (R version 3.1.0).

## Additional Information

**How to cite this article**: Feng, X. *et al.* Social correlates of the dominance rank and long-term cortisol levels in adolescent and adult male rhesus macaques (*Macaca mulatta*). *Sci. Rep.* **6**, 25431; doi: 10.1038/srep25431 (2016).

## Figures and Tables

**Table 1:** Correlation of stress-related behaviors with hair cortisol levels in adolescents.

Correlation with cortisol level	Receipt of (extreme and mild) aggression	Receipt of (extreme and mild) submission	(Extreme and mild) aggressive displays	(Extreme and mild) submissive displays
r	−0.160, −0.299	0.057, −0.379	−0.208, −0.127	0.057, −0.145
p	0.547, 0.304	0.154, 0.180	0.426, 0.660	0.154, 0.602

Spearman correlation.

**Table 2:** Correlation of stress-related behaviors with hair cortisol levels in adults.

Correlation with cortisol	Receipt of (extreme and mild) aggression	Receipt of (extreme and mild) submission	(Extreme and mild) aggressive displays	(Extreme and mild) submissive displays
r	0.686, 0.138	#, −0.506	0.025, 0.069	#, 0.385
p	0.025, 0.684	#, 0.111	0.826, 0.843	#, 0.244

(Spearman correlation, # means not available).
